# Optimized methods for obtaining sequencing-quality RNA from blubber of free-ranging cetaceans collected under field conditions

**DOI:** 10.1093/conphys/coag029

**Published:** 2026-04-22

**Authors:** Kelvin K A Boateng, Robyn F Allen, Randall S Wells, Nicholas M Kellar, Jane I Khudyakov

**Affiliations:** Department of Biological Sciences, University of the Pacific, 3601 Pacific Ave., Stockton, CA, 95211, USA; Sarasota Dolphin Research Program, Brookfield Zoo Chicago, c/o Mote Marine Laboratory, 1600 Ken Thompson Pkwy, Sarasota, FL, 34236, USA; Sarasota Dolphin Research Program, Brookfield Zoo Chicago, c/o Mote Marine Laboratory, 1600 Ken Thompson Pkwy, Sarasota, FL, 34236, USA; Marine Mammal and Turtle Division, Southwest Fisheries Science Center, National Oceanic and Atmospheric Administration, 8901 La Jolla Shores Dr., La Jolla, CA, 92037, USA; Department of Biological Sciences, University of the Pacific, 3601 Pacific Ave., Stockton, CA, 95211, USA

**Keywords:** Adipose, blubber, cetacean, dolphin, RNA, sequencing, transcriptome

## Abstract

Cetaceans (whales, porpoises and dolphins) play critical roles in marine ecosystems, but many populations are declining and are vulnerable to anthropogenic disturbance. Understanding the impacts of disturbance on the physiology and health of cetacean populations and developing robust methods of assessing them are critical for their conservation. Many current approaches for studying stress in cetaceans do not address the downstream impacts of stress hormones and contaminants, which mediate their effects by altering gene activity in target tissues. The latter can be examined by transcriptome sequencing, which can rapidly produce species- and tissue-specific global gene expression profiles that can be correlated with hormone and contaminant levels to identify markers of stress and pollutant exposure. However, transcriptome studies of cetacean blubber have been limited by the high lipid and structural fibre content of this tissue, which typically yields low-quality RNA that is not suitable for sequencing. In this study, we conducted a comprehensive comparison of tissue handling and RNA extraction methods for transcriptome studies of blubber collected from free-ranging cetaceans under field conditions. We subsampled blubber biopsies obtained from wild bottlenose dolphins during routine health assessments and compared the effect of sample preservation, tissue homogenization and choice of nucleic acid extraction kit on RNA yield and integrity. We found that flash-freezing blubber upon collection, homogenization using cryogenic milling followed by bead beating and RNA extraction using a phenol–guanidine–chloroform and silica spin column kit designed for fatty and fibrous tissues significantly improve RNA quality. Using the pipeline that we developed, we show that it is possible to obtain large yields of intact RNA across the full depth of dolphin blubber with integrity values that exceed those reported thus far (up to 8.3) and that are suitable for stress biomarker discovery by RNA sequencing, facilitating health assessments of wild cetaceans sampled by remote biopsy.

## Abbreviations

DEPC: diethylpyrocarbonate
RT-qPCR: reverse-transcription and real-time polymerase chain reaction
RIN: RNA integrity number
RNA: ribonucleic acid
RNAseq: RNA sequencing

## Introduction

Cetaceans (whales, porpoises and dolphins) play critical roles in marine ecosystems (Estes *et al.,* 2016; Kiszka *et al.,* 2022). Large whales, in particular, shape ecosystems by altering primary productivity and sequestering carbon (Pershing *et al.,* 2010; Roman *et al.,* 2014). However, one in four cetacean species is listed as threatened with extinction (Braulik *et al.,* 2023). In addition to ship strikes (Nisi *et al.,* 2024), cetacean health and survival is threatened by anthropogenic sound disturbance, contaminant exposure and climate change, among other sublethal factors (Desforges *et al.,* 2018; Duarte *et al.,* 2021; Kebke *et al.,* 2022). Understanding the impacts of these threats on physiology and health of cetacean populations and developing robust methods of assessing them are therefore critical for their conservation (National Academies of Sciences, Engineering, and Medicine, 2017).

Physiological studies of cetaceans are challenging due to their large sizes, fully aquatic lifestyles and often migratory life histories (Hunt *et al.,* 2013). Current approaches for assessing cetacean health include photogrammetry and measurement of lipid, hormone and contaminant levels in remotely sampled skin and blubber biopsies, as well as other matrices such as respiratory vapour and faeces (Hunt *et al.,* 2013; Castrillon and Bengtson Nash, 2020; Derous *et al.,* 2020; Sanganyado *et al.,* 2021; Van Cise *et al.,* 2024). While these approaches are extremely valuable, they do not address the downstream impacts of stress hormones and contaminants, which mediate their effects by altering the activity of cell-surface and nuclear receptors and expression of large clusters of genes (Rothhammer and Quintana, 2019; Jin *et al.,* 2025). The influence of anthropogenic stress on the latter can be examined by transcriptome sequencing, which produces species- and tissue-specific global gene expression profiles that can be correlated with hormone and contaminant levels and other measurements to identify gene markers of stress, pollutant exposure and, potentially, fitness (Williams *et al.,* 2011). Such markers can then be measured by targeted assays [i.e. reverse-transcription and real-time polymerase chain reaction (RT-qPCR)] of remotely collected biopsies from populations of concern to assess their stress state and inform conservation decisions. Importantly, gene expression in biopsies may serve as proxy for other measurements that are not feasible in large, free-ranging cetaceans, such as blood hormone levels, or that require specialized equipment and expertise, such as contaminant loads.

Numerous studies have examined the impact of environmental, physiological and behavioural variables on gene expression in cetacean skin and blubber tissues (Ball *et al.,* 2017; Trego *et al.,* 2019; Linsky *et al.,* 2024b). Most transcriptome studies of free-ranging cetaceans to date have used skin, which is easily obtained by remote dart biopsy (Van Dolah *et al.,* 2015; Neely *et al.,* 2018; Yoshimura *et al.,* 2024). While cetacean epidermis is highly vascularized, has high proliferative and transcriptional activity and may reflect contaminant accumulation in other organs (Trego *et al.,* 2019; Mancia *et al.,* 2021; Menon *et al.,* 2022), the skin transcriptome is potentially more responsive to external (e.g. temperature, radiation, salinity) than internal (i.e. stress state) conditions (Van Dolah *et al.,* 2015). In contrast, blubber is an excellent target for stress studies as it is the main energy depot in marine mammals, is both a source and target of endocrine signals and is a sink for lipophilic pollutants (Castrillon and Bengtson Nash, 2020; Maia *et al.,* 2025). However, most blubber gene expression studies in cetaceans published to date have used targeted (RT-qPCR) rather than global (RNAseq) assays (Ball *et al.,* 2017; Linsky *et al.,* 2022; Linsky *et al.,* 2024a), limiting the discovery of novel biomarkers. RNA isolated from cetacean blubber has lower quality than RNA isolated from skin (Funasaka *et al.,* 2024), with integrity values below those recommended for transcriptome sequencing (Gallego Romero *et al.,* 2014). This is likely due to the high lipid and structural fibre content of cetacean blubber (Montie *et al.,* 2008), which may interfere with tissue preservation and homogenization. Two recent studies described the blubber transcriptomes of odontocetes managed under human care (Suzuki *et al.,* 2024a; Suzuki *et al.,* 2024b), but the sample processing protocols likely differed from field conditions under which wild cetaceans are usually sampled. Therefore, improvement of tissue handling and RNA purification approaches is needed to accelerate biomarker discovery.

A number of methods have been developed for RNA extraction from tissues high in protein fibre, lipid and/or RNase content (Nouvel *et al.,* 2021). A typical RNA isolation protocol involves manual or automated (e.g. bead mill) disruption of tissue in lysis buffer followed by phenol–guanidinium–chloroform phase extraction (Chomczynski and Sacchi, 2006) and RNA purification using silica spin columns. Some tissues require specialized approaches. For example, incubation of field-sampled and/or RNase-rich tissues in an ammonium sulphate solution (e.g. RNA*later*^®^) immediately after collection may be necessary to stabilize RNA and protect it from degradation (Wang *et al.,* 2018). Isolation of RNA from very fibrous tissues may require pulverization in liquid nitrogen to achieve complete homogenization (Le Bleu *et al.,* 2017; Takács *et al.,* 2023). RNA extraction from lipid-rich tissues may require the use of reagents and kits developed specifically for this purpose (Nandi Jui *et al.,* 2022). Studies of blubber gene expression to date have used different approaches (Ball *et al.,* 2017; Linsky *et al.,* 2022; Suzuki *et al.,* 2024b), and systematic evaluation of methods for (1) tissue preservation, (2) tissue homogenization and (3) extraction of high-quality RNA is currently lacking for cetacean blubber, hindering biomarker discovery.

In this study, we conducted a comprehensive comparison of blubber handling and RNA extraction methods for transcriptome studies of blubber collected from free-ranging cetaceans under field conditions. We subsampled blubber biopsies obtained from wild common bottlenose dolphins (*Tursiops truncatus*) during routine health assessments and compared the effects of sample preservation, tissue homogenization and choice of nucleic extraction kit on RNA yield and integrity. Using the pipeline that we developed, we show that it is possible to obtain large yields of intact RNA across the full depth of *Tursiops* blubber with integrity values that exceed those reported thus far and that are suitable for RNA sequencing. This advancement in methodology will facilitate transcriptome-wide studies of cetacean blubber, which have high potential for identifying novel biomarkers of stress and health in free-ranging cetaceans, as has been shown previously in pinnipeds (Deyarmin *et al.,* 2019; Pujade Busqueta *et al.,* 2020).

## Materials and Methods

### Ethical declarations

Animal sampling was conducted under National Marine Fisheries Service MMPA Scientific Research Permit No. 20455 and with approval of the Institutional Animal Care and Use Committee at Mote Marine Laboratory. All animal handling was performed in accordance with the relevant guidelines and regulations.

### Sample collection

**Figure 1:**
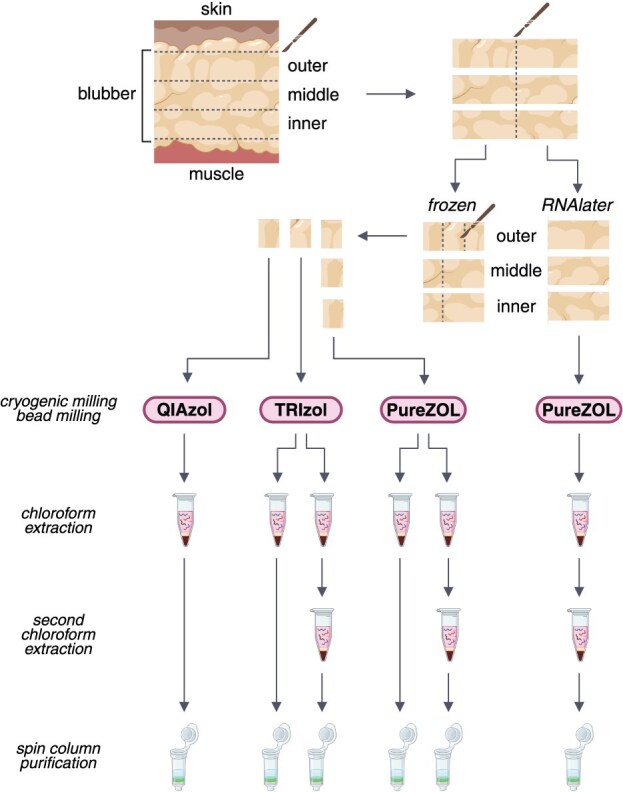
Schematic of the study design. Full-depth blubber biopsies were obtained during health assessments of free-ranging *Tursiops* (*n* = 6). To determine whether vertical stratification in fat and structural fibre content affects RNA extraction from blubber, biopsies were subsampled horizontally into three layers upon collection. To compare the impacts of sample preservation on RNA quality, each layer was bisected in two, and one half was preserved using RNA*later* reagent while the other half was flash-frozen in liquid nitrogen. To determine the effect of RNA isolation kit on RNA quality, flash-frozen outer blubber was further subsampled and homogenized using either TRIzol, QIAzol or PureZOL lysis reagent. After phase extraction with chloroform, samples lysed using TRIzol and PureZOL underwent a second phase extraction, after which RNA extracted from all samples was purified using spin columns. Created in BioRender. Khudyakov, J. (2026), https://BioRender.com/71gevtm.

**Figure 2:**
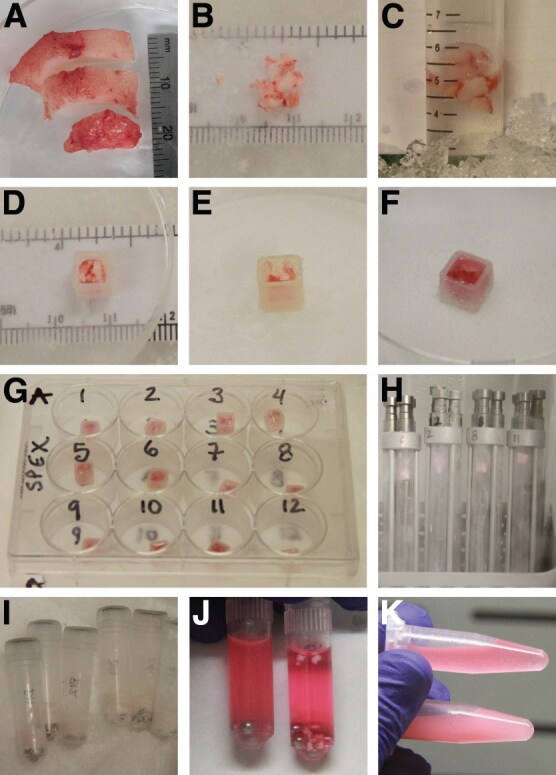
Illustration of *Tursiops* blubber sample homogenization methods. (**A**) Blubber biopsies were separated into outer (top of image), middle and inner layers prior to processing. (**B**) Blubber tissue was minced on dry ice, either prior to overnight incubation in RNA*later* reagent (**C**) or after flash-freezing, prior to homogenization. (**D** and **E**) Frozen, minced blubber tissue pieces were packed into silicone cube moulds on dry ice. (**F**) Lysis solution such as TRIzol was used to fill the remaining space in the mould. (**G**) Cube moulds were frozen and stored in multi-well plates. (**H**) Frozen tissue cubes were loaded into cryogenic grinding microvials in liquid nitrogen for processing. (**I**) Pulverized tissue was transferred into bead tubes for further homogenization by bead milling. (**J** and **K**) Cryogenic grinding of tissue prior to bead beating (left tube in J, bottom tube in K) resulted in more effective homogenization than bead milling alone (right tube in J, top tube in K).

Blubber samples were collected during catch-and-release health assessments of *T. truncatus* in shallow waters in Sarasota Bay, FL, by the Brookfield Zoo Chicago’s Sarasota Dolphin Research Program, as described previously (Wells *et al.,* 2004; Sherman *et al.,* 2021; Wells *et al.,* 2025). Full-depth blubber biopsies were surgically excised by veterinary staff after sterilizing the biopsy site with chlorhexidine solution as well as methanol and injecting an anaesthetic solution consisting of 2% lidocaine with epinephrine. Samples from six *Tursiops* individuals were used for this study. Blubber biopsies were placed in sterilized glass or plastic Petri dishes on cold packs or dry ice within 10 min of collection. All tools used for tissue handling and dissection were wiped with RNase*Zap*™ solution (Ambion, USA) followed by 70% alcohol in diethylpyrocarbonate (DEPC)-treated water. After separating pigmented epidermis from underlying blubber, a full-depth subsample of blubber was obtained from each biopsy using a sterile scalpel. To assess the potential impact of vertical stratification of lipid and structural fibre content (Montie *et al.,* 2008) on RNA extraction, blubber was vertically subdivided into three layers, each approximately 6–8 mm deep: outer (dorsal, closest to epidermis), middle and inner (ventral, closest to muscle; Figs 1 and 2A). Each layer was further bisected in two, and one half was minced and placed into a 5-ml cryovial filled with RNA*later*^®^ reagent (Ambion, USA) on wet ice (Figs 1 and 2B and C), while the other was placed in a 2-ml cryovial and into a cryogenic storage dewar with liquid nitrogen. Samples were frozen or placed in RNA*later* within 30 min of biopsy excision. Samples in RNA*later* were kept on wet ice or a 4°C fridge overnight, after which the liquid was removed, and tissue was frozen on dry ice or in a −80°C freezer. Samples were stored at −80°C and transported between laboratories on dry ice.

### Blubber homogenization

Reagents and materials were purchased from VWR or Fisher Scientific, unless otherwise indicated. All non-consumable supplies used for blubber processing were treated with RNase*Zap*™ as described above. Blubber samples were subsampled for different RNA extraction protocols in the laboratory (Fig. 1) using a sterile scalpel in a plastic Petri dish on dry ice. Subsamples were weighed and minced into 1- to 2-mm pieces (Fig. 2B). Minced blubber tissue (45–80 mg) was placed either directly into a 2.0-ml tube containing 3.175-mm-diameter metal beads (Lysing Matrix S, MP Biomedicals, USA) or into a 5-mm silicone cube mould for making resin jewellery kept on dry ice (Etsy, USA; Fig. 2D and E).

We compared the integrity and yield of RNA isolated from blubber using three different lysis reagents: TRIzol™, QIAzol™ and PureZOL™ (Ambion, Qiagen and Bio-Rad, respectively; Fig. 1). We also assessed the effect of including an additional phase extraction step during RNA isolation with TRIzol and PureZOL, and the impact of silica spin column-based RNA cleanup on the quality of TRIzol-extracted RNA. For homogenization by bead milling, 1 ml of lysis reagent was added to each bead tube and tubes were processed for two to three cycles in either the Bullet Blender^®^ Tissue Homogenizer (Next Advance, Inc., USA; each cycle: 2 min at power 12) or TissueLyser LT Compact Bead Mill (Qiagen, USA; each cycle: 2 min at 50 Hz). Tissue homogenization appeared to be more effective with the Bullet Blender compared to TissueLyser (data not shown), so the former was used for all subsequent processing.

For homogenization by cryogenic milling (cryomilling), lysis reagent (20–50 μl; TRIzol, QIAzol or PureZOL) was used to fill the space remaining in the mould after adding tissue and frozen on dry ice (Fig. 2F). Tissue cubes were stored in 12-well plates (Fig. 2G) on dry ice or at −80°C until processing. Frozen cubes were removed from moulds using forceps and placed into pre-cooled cryogenic grinding microvials (Fig. 2H; Microvial Grinding Set, Cat. No. 6757, Cole-Palmer, USA). Tissue was pulverized in liquid nitrogen using the SPEX SamplePrep 6875 Freezer/Mill High-Capacity Cryogenic Grinder (Cat. No. 6875-115, Cole-Palmer, USA) with the ‘rubber’ protocol (15-min pre-cooling, followed by three cycles of 2-min grinding at 15 cps with 2 min of cooling between cycles) to produce fully powdered tissue (Fig. 2I).

Cryomilled tissue powder was transferred from grinding vials to bead tubes, followed by addition of 1 ml of the appropriate lysis reagent. Bead milling was conducted for two to three rounds as described above. Tissue homogenates were further disrupted by passage through a 22G needle and 1-ml syringe, centrifuged for 10 min at 12 000 × *g* to pellet insoluble cellular material and transferred to clean tubes. Homogenates were stored at −80°C if not processed the same day.

### RNA isolation

Tissue homogenates (approximately 1 ml) were combined with 200 μl of chloroform to obtain RNA using phase separation. For double extraction protocols (‘TRIzol-2’ and ‘PureZOL-2’ methods), the aqueous phase from the first extraction was brought up to 1-ml total volume with lysis reagent, combined with another 200 μl of chloroform, and phase separation was repeated. For the TRIzol method, RNA was precipitated from the aqueous phase with isopropanol and 20 μg of glycogen, pelleted by centrifugation, washed with 75% ethanol and resuspended in 30 μl of DEPC-treated water. TRIzol-extracted RNA samples undergoing spin column purification with the RNeasy™ Mini Kit (Qiagen, USA; ‘TRIzol-col’ Methods) were combined with 350-μl RLT reagent supplemented with 1:100 beta-mercaptoethanol and 250-μl ethanol prior to column purification following the manufacturer’s protocol. For the ‘QIAzol’ and ‘PureZOL’ methods, RNA was precipitated from the aqueous phase and purified on columns using the RNeasy™ Lipid Mini Kit (Qiagen, USA) and Aurum™ Total RNA Fatty and Fibrous Tissue Kit (Bio-Rad, USA), respectively, following the manufacturers’ protocols. All spin column methods included a 15-min digest with DNase I enzyme provided in the kits. Samples were eluted in 30–50 μl of DEPC-treated water.

### RNA quality assessment

RNA quantity was determined using the Qubit™ Broad Range RNA Quantification kit (Invitrogen, USA). RNA yield was determined by dividing the amount (ng) of RNA by the mass of blubber tissue used for homogenization. Yields were not calculated for the PureZOL-2 protocol as frozen tissue mass was not recorded. RNA integrity was assessed using the RNA 6000 Pico Kit on the 2100 Bioanalyzer instrument (Agilent Technologies, Inc., USA) after diluting samples to 1–5 ng/μl concentration in DEPC-treated water. We used a subset of samples to calculate reproducibility of the Qubit (*n* = 6 samples) and Bioanalyzer (*n* = 3 samples) assays. The intra- and inter-assay coefficients of variation (CVs) for the Qubit kit were <3% and <6%, respectively. The intra-assay and inter-assay CVs for the Bioanalyzer kit were <3% and <10%, respectively. The remaining samples were assayed without technical replicates.

### Statistical analyses

All statistical analyses were conducted using R software v4.5.1 run in RStudio v2025.09.1+401. Linear mixed-effects (LME) models were used to determine the effect of the RNA extraction method on RNA yield or integrity [as measured by RNA integrity number (RIN)] with animal from which blubber was obtained as a random effect and extraction method as a fixed effect using the lme4 package (Bates *et al.,* 2015). Post hoc tests using estimated marginal means were used for pairwise comparisons between methods with the emmeans package, with Benjamini–Hochberg correction of *P* values for multiple hypothesis testing (Searle *et al.,* 1980). LMEs were also used to examine the effect of the sample preservation method or blubber layer on RNA integrity. Model residuals were visually assessed for homoscedasticity and normality using Q–Q plots and confirmed by Breusch–Pagan and Shapiro–Wilk tests, respectively. Levene’s test was used to compare variances in RNA metrics between methods, which did not differ for either RINs (*P* = 0.42) or yield (*P* = 0.74). The per-sample cost of RNA extraction reagents was determined using manufacturer list prices in November 2025.

## Results

### Tissue homogenization

Bead milling alone did not fully disrupt *Tursiops* blubber samples, as intact tissue pieces remained even after three cycles of homogenization with either of two bead mill instruments (Fig. 2J). In contrast, cryomilling followed by bead beating produced opaque homogenates that did not have any visible tissue particles (Fig. 2J and K). However, cryomilling required substantial optimization. While intact cubes of 80–100 mg of blubber were not effectively pulverized by multiple cycles of cryomilling (data not shown), smaller minced pieces (Fig. 2B) evaded impactor rods in cryogenic vials. Therefore, we followed the suggestion of the cryomill manufacturer to freeze minced tissue pieces (40–70 mg) in an ‘ice cube’ with lysis reagent prior to processing (Fig. 2D–H). Complete pulverization of tissue-reagent cubes was obtained after one to two rounds of cryomilling.

### Tissue preservation

**Figure 3:**
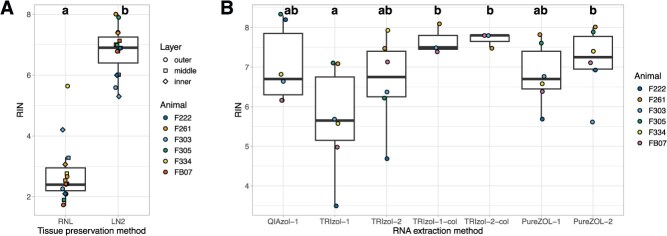
The choice of tissue preservation approach and RNA isolation kit affect the integrity of RNA extracted from *Tursiops* blubber. (**A**) RNA integrity numbers (RINs) of outer, middle and inner blubber samples (*n* = 6 animals) preserved by either flash-freezing or overnight incubation with RNA*later* after collection. RNA*later* treatment significantly decreased RNA integrity (*F*_1,23_ = 167.62, *P* < 0.0001), while RINs did not differ between blubber layers (adjusted *P* = 0.74). (**B**) RINs of RNA extracted from flash-frozen outer blubber samples (*n* = 6 animals) varied by kit and method used for isolation (*F*_6,24_ = 2.97, adjusted *P* = 0.026). Different letters denote groups that differed significantly (adjusted *P* < 0.05) by RIN. QIAzol-1: RNeasy Lipid Mini Kit with a single chloroform extraction. TRIzol-1 and TRIzol-2: TRIzol RNA extraction with one or two chloroform extractions, respectively, and no column cleanup or DNase I digest. TRIzol-1-col and TRIzol-2-col: TRIzol RNA extraction with one or two chloroform extractions, respectively (*n* = 3 animals), and RNeasy Mini Kit cleanup. PureZOL-1 and PureZOL-2: Aurum Total RNA Fatty and Fibrous Tissue Kit with one or two chloroform extractions, respectively.

We extracted RNA from *Tursiops* blubber samples that were vertically subsampled into three layers (outer, middle, inner; Figs 1 and 2A) and either flash-frozen or treated with RNA*later* reagent upon collection. Preservation of blubber in RNA*later* significantly decreased RNA integrity compared to flash-freezing (*F*_1,23_ = 167.62, *P* < 0.0001). Mean RIN of flash-frozen samples was 4.0 units higher than that of samples preserved in RNA*later* (Fig. 3A and Table 1). RNA integrity did not vary significantly between outer, middle and inner blubber layers with either preservation method (adjusted *P* = 0.74; Fig. 3A and Table 1).

**Table 1:** Quality of RNA extracted from *T. truncatus* blubber as measured by microcapillary gel electrophoresis and RIN (ranging 1–10 from most degraded to most intact)

	***N***	**Mean (SD) yield (ng/mg)**	**Mean (SD) RIN**	**Max RIN**	**Min RIN**
Blubber preservation method					
RNA*later*^a^	15	—	2.77 (1.00)	5.60	1.70
Flash-frozen^a^	15	—	6.81 (0.78)	8.00	5.30
Blubber layer					
Outer blubber^a^^,^^b^	6	—	7.10 (0.88)	8.00	5.60
Middle blubber^a^^,^^b^	6	—	6.80 (0.40)	7.10	6.00
Inner blubber^a^^,^^b^	3	—	6.23 (1.07)	7.40	5.30
RNA isolation method					
QIAzol (one-phase extraction method)^c^	6	127.8 (59.5)	7.05 (0.96)	8.30	6.20
PureZOL (one-phase extraction method)^c^	6	110.5 (41.4)	6.82 (0.78)	7.80	5.70
PureZOL (two-phase extraction method)^c^	6	—	7.15 (0.87)	8.00	5.60
TRIzol (one-phase extraction method)^c^	6	178.0 (98.1)	5.67 (1.36)	7.10	3.50
TRIzol (one-phase extraction method + column)^c^	3	144.3 (74.7)	7.67 (0.38)	8.10	7.40
TRIzol (two-phase extraction method)^c^	6	94.5 (85.0)	6.63 (1.14)	7.90	4.70
TRIzol (two-phase extraction method + column)^c^	3	101.4 (35.6)	7.70 (0.17)	7.80	7.50

RNA yield data were not obtained for samples that were not weighed prior to processing. *N* refers to the total number of subsamples of blubber from *Tursiops* individuals (*n* = 6) that were processed for each method.

a ^a^PureZOL two-phase extraction method.

b ^b^Flash-frozen blubber.

c ^c^Outer blubber layer (flash-frozen).

### Tissue lysis and RNA extraction

**Figure 4:**
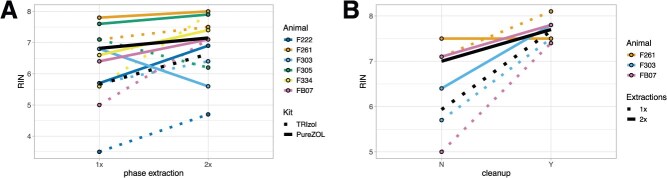
(**A**) The addition of a second chloroform phase extraction step to the TRIzol (dotted lines) and PureZOL (solid lines) protocols caused a modest but not significant (*P* > 0.05) increase in quality of RNA isolated from *Tursiops* blubber (*n* = 6 animals). Black lines show mean RIN values for each protocol. (**B**) RNA isolated using the TRIzol method with either one chloroform phase extraction (dotted lines) or two chloroform phase extractions (solid lines; *n* = 3 animals) showed a significant improvement in RINs after silica spin column-based kit cleanup (*P* = 0.039). 1x and 2x denote one and two chloroform phase extractions, respectively. N and Y denote samples before and after column cleanup, respectively.

**Figure 5:**
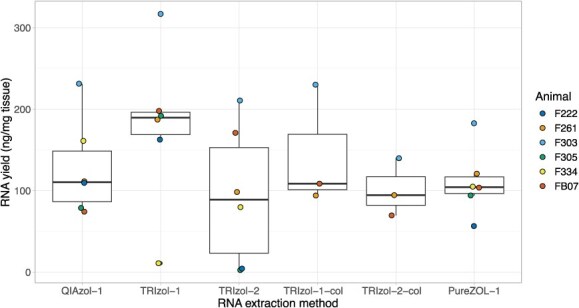
Microcapillary gel electropherograms of RNA isolated from subsamples of outer blubber tissue from one *Tursiops* individual (F261). Panel **A** shows RNA isolated using the PureZOL-2 method from tissue that was preserved in RNA*later*. Panels **B**–**F** show RNA that was isolated from flash-frozen tissue using (B) TRIzol-1, (C) TRIzol-1-col, (D) QIAzol, (E) PureZOL-1 and (F) PureZOL-2 methods.

The choice of lysis reagent and extraction kit used for purification significantly influenced RNA quality (*F*_6,24_ = 2.97, *P* = 0.026; Fig. 3B and Table 1). Specifically, the integrity of RNA isolated using TRIzol with a single chloroform extraction step and no silica column cleanup was lower than that of RNA isolated using other column-based methods (adjusted *P* < 0.05). Significantly, RNA isolated using two chloroform extraction steps and purified using a silica column-based kit had RINs >7, which is considered the quality threshold for RNA sequencing (Fig. 4). Electropherograms of RNA extracted from a representative outer blubber sample using different methods are shown in Fig. 5.

**Figure 6:**
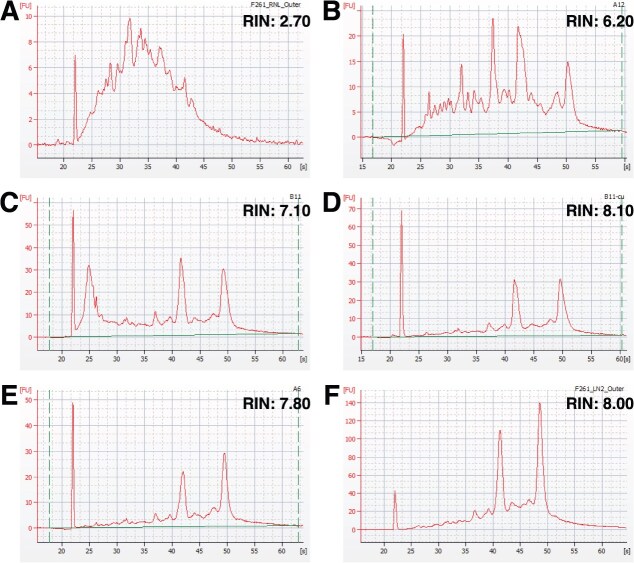
Choice of RNA isolation kit had a marginally significant impact on RNA yield from *Tursiops* blubber (*n* = 6 animals; nanograms of RNA per milligram of frozen tissue; *F*_5,19_ = 2.58, adjusted *P* = 0.060). Yields were not calculated for the PureZOL-2 protocol as frozen tissue mass was not recorded.

The choice of RNA extraction protocol had a marginally significant effect on RNA yield (nanograms of RNA per milligram of frozen tissue; *F*_5,19_ = 2.58, *P* = 0.060; Fig. 6 and Table 1). RNA yields trended higher for samples processed using TRIzol with a single chloroform extraction and no silica column cleanup (adjusted *P* = 0.068). Mean RNA yield for all methods using a single chloroform extraction step and silica columns was 124.2 ± 53.3 ng of RNA per milligram of frozen tissue.

## Discussion

In this study, we demonstrate that isolation of intact, high-quality RNA from cetacean blubber requires extensive tissue homogenization that is facilitated by cryomilling. In contrast, phocid blubber collected and frozen in the field homogenizes easily with bead beating and routinely yields highly intact RNA (e.g. mean 8.2 ± 0.4 across full blubber depth; Khudyakov *et al.,* 2022). This difference is likely due to the higher content of structural protein fibres in cetacean compared to pinniped blubber (Montie *et al.,* 2008). Therefore, full disruption of tissue requires an approach similar to that used with other highly fibrous connective tissues, such as cartilage (Le Bleu *et al.,* 2017; Takács *et al.,* 2023). Our pipeline involved mincing blubber into 1- to 2-mm pieces on dry ice, freezing the pieces with lysis reagent in a 5-mm cube mould, cryomilling the frozen tissue cubes into a powder, bead milling the powder in lysis reagent and shearing genomic DNA with a needle and syringe. While this process is labor intensive, it can be performed in stages over multiple days, as tissue cubes, cryomilled tissue powder and bead-milled tissue homogenates can be stored at −80°C until further processing. Importantly, the sample pulverization protocol produced consistent yields and quality of RNA across blubber depth, despite the well-described vertical stratification in fibrous and lipid content in cetacean blubber (Montie *et al.,* 2008). This suggests that the difference in fibrous content between different layers within cetacean blubber is less significant than the difference in fibrous content between cetacean and pinniped blubber. Overall, we show that this homogenization approach produces mean RNA yields of >100 ng/mg tissue (max: 317 ng/mg) from small amounts of blubber (≥50 mg) with RINs >6.0 (max: 8.3) for all phenol–guanidinium thiocyanate–chloroform-based RNA isolation approaches that we tested (see Supplementary File 1 for protocol).

Our study confirmed that flash-freezing cetacean blubber in the field is preferable to tissue preservation in RNA*later*, a high ionic strength salt solution developed for stabilization of RNA in tissue samples (Hemmrich *et al.,* 2010). In contrast, RNA*later* treatment of phocid blubber does not affect RNA quality (Pujade Busqueta *et al.,* 2020). The RNA*later* protocol requires tissue refrigeration overnight (0–4°C) for the reagent to fully permeate the tissue. If tissue is stored at higher temperatures or permeation is incomplete because tissue pieces are too large, lipid-rich or very dense, RNA can potentially degrade during that time (Gayral *et al.,* 2011; Jones and Kennedy, 2015; Wang *et al.,* 2018; Passow *et al.,* 2019). RNA*later* also makes tissue more rubbery and difficult to homogenize (Nouvel *et al.,* 2021), which may cause incomplete lysis and decrease the resulting RNA quality. While RNA*later* has been shown to stabilize transcriptome profiles at the time of collection in cultured cells, treatment of complex tissue samples with this reagent alters gene expression in a non-random manner (van Eijsden *et al.,* 2013; Passow *et al.,* 2019). Therefore, we recommend freezing tissue in the field, unless there is potential for it to thaw during transport or storage (in which case, RNA*later* may reduce RNA degradation at warmer temperatures). Our preliminary tests showed that RNA integrity did not differ between tissue frozen on dry ice versus liquid nitrogen, despite slower freezing rates with the former (data not shown). For researchers sampling cetaceans in challenging field conditions, and those that do not have access to liquid nitrogen, freezing blubber samples in a styrofoam cooler on dry ice may offer a more practical approach for sample processing and storage. Keeping tissue on dry ice during processing also makes it firmer and easier to dissect.

Finally, we conducted an in-depth comparison of three commonly used RNA isolation kits and modifications to the associated protocols to identify those producing the most intact RNA from cetacean blubber. RNA isolation from biological tissues using the TRIzol reagent is the most commonly used and simplest approach (Chomczynski and Sacchi, 2006; Rio *et al.,* 2010). TRIzol produced marginally higher yields of RNA, but its quality was significantly lower than the other kits we tested. RNA quality could be improved by either (1) including a second chloroform phase extraction step during the protocol and/or (2) cleaning up the TRIzol-extracted RNA using a silica spin column-based kit (Lee *et al.,* 2015). However, both amendments led to RNA loss. In our trials, a second chloroform extraction only marginally improved RNA quality on its own, while column-based cleanup of TRIzol-isolated RNA significantly increased RNA integrity, regardless of the number of chloroform extractions. Therefore, for researchers working with limited tissue quantities and using the TRIzol method, we recommend a single extraction and column cleanup. The column cleanup protocol includes a fast and convenient step for digesting genomic DNA, which can bias gene expression analyses if it is not removed (Li *et al.,* 2022).

Since the publication of the phenol–guanidine thiocyanate–chloroform protocol of RNA extraction (Chomczynski and Sacchi, 1987), this method has been adapted by commercial kit manufacturers for many applications, including small input tissue amounts and lipid-rich and/or fibrous tissues. Kits designed for lipid-rich tissues reduce RNA loss by integrating silica spin column purification directly into the protocol (Nandi Jui *et al.,* 2022). We routinely use the RNeasy Lipid Mini kit with QIAzol to isolate sequencing-quality RNA from phocid blubber (Khudyakov *et al.,* 2022). In this study, we found that the QIAzol method produced RNA yields and quality from cetacean blubber that did not differ significantly from the other column-based methods, although it did yield the highest maximum RIN (8.3) of all methods tested.

Due to the high structural fibre content of cetacean blubber, we also tested kits specifically developed for fibrous tissues. However, our preliminary tests using Qiagen’s RNeasy Fibrous Tissue Mini Kit, which uses a buffer with beta-mercaptoethanol rather than phenol–guanidine for tissue lysis, did not yield detectable quantities of RNA (data not shown). Therefore, we tested a kit that was developed for tissues that are both fibrous and lipid-rich (PureZOL), and which has been used for gene expression studies of cetacean skin (Panti *et al.,* 2022; Yoshimura *et al.,* 2024). The PureZOL kit produced high yields of intact RNA that were on par with the QIAzol kit and TRIzol with column cleanup. Moreover, adding a second chloroform extraction to the protocol marginally improved the quality of isolated RNA. While not significant, the variance in RIN values between samples isolated with the PureZOL kit (0.61) was lower than that of QIAzol (0.92), suggesting better consistency in RNA extractions. Ultimately, the choice of RNA extraction kit may be influenced by cost. Apart from the TRIzol method without silica column cleanup ($2.54 per sample), the PureZOL method was more cost-effective ($10.36 per sample) compared to both TRIzol with column cleanup ($14.52 per sample) and QIAzol ($15.26).

In summary, we show that large yields (e.g. >10 μg from 60 mg of tissue) of highly intact, sequencing-quality RNA can be obtained from cetacean blubber RNA samples collected, processed and frozen in field conditions, including long boat days in >30°C weather and full sun. To our knowledge, this is the first study to conduct comprehensive, within-sample comparison of the effects of RNA extraction protocols on the quality of cetacean blubber RNA. Surgically collected biopsies from a cetacean species that is routinely monitored via health assessments enabled us to obtain sufficient tissue amounts for subsampling and extensive method comparisons. However, the broad applicability of the reported methods should be tested in further studies. For example, this protocol should be validated using tissue from other cetacean species, such as those with extremely high lipid and structural fibre contents (e.g. capital breeding mysticetes), and in blubber sampled under different field conditions, such as via biopsy dart, which exposes tissue to seawater during collection. Nevertheless, the optimized protocol provided in Supplementary File 1 offers a starting point for transcriptome-wide studies of blubber responses to physiological and environmental stressors that can be used to identify gene markers of stress and health in cetaceans.

## Supplementary Material

Web_Material_coag029

## Data Availability

Raw data are available on Figshare (doi:10.6084/m9.figshare.30716306).
