# Supplementary material for: Optimized methods for obtaining sequencing-quality RNA from blubber of free-ranging cetaceans collected under field conditions
Source: Conserv Physiol. 2026 Apr 22;14(1):coag029. doi: 10.1093/conphys/coag029 (PMC13102499; doi:10.1093/conphys/coag029)
Supplement: Web_Material_coag029 [file web_material_coag029.zip › Supplementary File 1.docx]

**Optimized methods for obtaining sequencing-quality RNA from blubber of free-ranging cetaceans collected under field conditions**

Kelvin K. A. Boateng^1^, Robyn F. Allen^2^, Randall S. Wells^2^, Nicholas M. Kellar^3^, Jane I. Khudyakov^1^

^1^ Department of Biological Sciences, University of the Pacific, Stockton, CA, USA

^2^ Sarasota Dolphin Research Program, Brookfield Zoo Chicago, c/o Mote Marine Laboratory, Sarasota, FL, USA

^3^ Marine Mammal and Turtle Division, Southwest Fisheries Science Center, National Oceanic and Atmospheric Administration, La Jolla, CA, USA

**PROTOCOL: RNA Extraction from Cetacean Blubber**

- To protect samples from RNA degradation by RNase enzymes, always wear gloves, use consumables labeled as sterile and "nuclease-free" and water treated with diethyl pyrocarbonate (DEPC), and wipe all working surfaces, dissection tools, pipettor barrels, etc. with an RNase decontamination solution such as RNase*Zap*™ or RNase AWAY™.

**A. Sample collection and storage**

1. Chill a sterilized glass or sterile plastic petri dish on a frozen cold pack or dry ice. Clean forceps with RNase decontamination solution and prepare a sterile scalpel.
2. Extract blubber tissue from biopsy dart using forceps and place in petri dish. Blot to remove any blood using sterile gauze or Kimwipes. Subsample with scalpel if needed.
3. Place tissue into sterile cryovial(s) and freeze as quickly as possible in a cooler with dry ice, a dewar containing liquid nitrogen, or a charged dry shipper.
4. Keep blubber continuously frozen until samples can be transferred to a -80°C freezer for long-term storage.
5. If dry ice or liquid nitrogen is unavailable, or if tissue cannot be continuously frozen, mince blubber into small (<5 mm) pieces and place in a cryovial with at least 1 ml of RNA*later*^®^*,* RNAprotect^®^, or similar stabilizing reagent for every 100 mg (~5 mm cube) of tissue. Keep on wet ice or in a 4°C fridge overnight, then remove liquid with a sterile transfer pipette (or transfer tissue with forceps to a clean and dry tube) and freeze at -20°C to -80°C. Blubber RNA quality may be decreased with RNA*later*^®^.

**B. Sample preparation for cryogenic grinding**

1. Chill a sterile plastic petri dish and silicone sample molds (pre-cleaned with RNase decontamination solution followed by 70% alcohol in DEPC water) on dry ice. Clean forceps with RNase decontamination solution and prepare a sterile scalpel.
2. Add ~20 μl of lysis reagent (PureZOL^®^ or QIAzol^®^) to the sample mold to cover the bottom with a thin layer. Allow solution to freeze on dry ice.
3. Transfer frozen sample to the cold petri dish. Weigh out 40-80 mg of tissue, mince into 1-2 mm pieces and pack tightly into the frozen sample mold.
4. Add lysis reagent (usually <50 μl) to fill any remaining space in the mold and to cover the sample pieces with a smooth layer of liquid. Do not overfill. Allow to freeze for at least one hour on dry ice or in a -80°C freezer. Filled molds with different samples can be organized in labeled multi-well culture plates and stored at -80°C until processing.
5. Place one end plug and one impactor rod into each cryogenic grinding microvial to be used for sample processing. Place microvials, capped end down, in a rack in a styrofoam container containing dry ice or a small amount of liquid nitrogen (2-4 cm deep).
6. Remove sample cubes from molds using forceps and place each cube into its pre-chilled microvial. Immediately add the second end plug (this may be challenging since plastic tubes shrink when frozen). Filled microvials can be kept in the styrofoam container if they will be processed the same day, or stored in a -80°C freezer until processing.

**C. Sample pulverization**

1. Fill cryogenic mill instrument with liquid nitrogen as recommended by the manufacturer.
2. Add cryogenic vials containing samples to the instrument. Pulverize using the "rubber" protocol: 15-minute pre-cool, followed by three cycles of two-minute grinding at 15 cps with two minutes of cooling between cycles.
3. Extract vials from the grinder and check that no intact tissue pieces remain. Uncap vials and dislodge any intact pieces using forceps, then repeat the protocol if necessary.
4. Keep microvials with tissue powder in the styrofoam container with liquid nitrogen or dry ice while processing or place in a -80°C freezer for longer-term storage.
5. Chill labeled 2-ml tubes containing metal beads on dry ice. Remove end caps from cryogenic grinding microvials using the cap extraction tool and transfer tissue powder to cold bead tubes. Store bead tubes with tissue powder on dry ice or in a -80°C freezer.

**D. Sample homogenization**

1. Add 1 ml of cold (~4°C) lysis reagent to each bead tube containing tissue powder. Process in a bead mill using two to three cycles of two-min bead beating at high speed. Homogenates should appear opaque with no visible tissue pieces or particles remaining. Chill samples on wet ice between bead beating cycles to avoid overheating.
2. Pass homogenates several times through a 22- or 23-G sterile needle and 1-ml syringe to shear genomic DNA. Use the needle and syringe to transfer homogenates to clean, RNase-free microcentrifuge tubes and let sit for 5 minutes at room temperature.
3. Centrifuge homogenates at 12,000 x g and 4°C for 10 minutes. Transfer cleared homogenates (pink color) to clean microcentrifuge tubes, making sure to avoid pellets of insoluble debris at the bottom and the clear lipid layer on top. Homogenates can be stored at -80°C until further processing.

**E. Phase extraction and RNA purification**

1. Allow tissue homogenates to thaw on wet ice if previously frozen. Add 200 μl of chloroform (molecular biology or biotechnology grade) to each tube and shake vigorously to mix. Let sit for 5 minutes at room temperature with occasional shaking.
2. Centrifuge homogenates at 12,000 x g and 4°C for 15 minutes. Carefully transfer the aqueous phase, which should appear clear or slightly yellow, to clean microcentrifuge tubes, being careful to avoid the white interphase and pink organic phase.
3. If proceeding with a second phase extraction, add an equal volume (usually 500-600 μl) of chloroform to the aqueous phase from the first extraction and repeat steps E1 and E2.
4. Add an equal volume of 70% ethanol (prepared with DEPC-treated water) to the aqueous phase, mix, and proceed with spin column purification as specified by the manufacturer (e.g., Bio-Rad Aurum™ Total RNA Fatty and Fibrous Tissue Kit Manual or Qiagen RNeasy^®^ Lipid Tissue Handbook). Include a 15-minute on-column DNase I digest step.
5. Elute RNA in 30-50 μl of DEPC-treated water or elution buffer provided with the RNA extraction kit. RNA samples can be concentrated using a second elution by adding the first eluate back to the column and re-centrifuging.
6. Determine RNA concentration using an RNA-specific fluorometric assay such as Qubit™ Broad-Range RNA Assay Kit. RNA integrity can be assessed by microcapillary gel electrophoresis (e.g., using Agilent Bioanalyzer with RNA 6000 Nano or Pico kit).

**Recommended materials and supplies**

- Cryogenic grinder such as SamplePrep Freezer/Mill (e.g., Cole-Parmer^®^ cat # 6875-115 / EW-04500-40)
- Grinding microvial set (0.1-0.5 ml polycarbonate vials with stainless steel end plugs and impactors; e.g., Cole-Parmer^®^ cat # 6757 / EW-61043-06)
- Multi-vial holder for grinding microvials (e.g., Cole-Parmer^®^ cat # 6807 / EW-04500-51)
- End plug extractor for grinding microvials (e.g., Cole-Parmer^®^ cat # 6758 / EW-04500-44)
- Recommended: 30 L liquid nitrogen dewar with a manual discharge device for filling cryomill (e.g., MVE LAB 30 liquid nitrogen system), unless the cryogenic mill is located near a large liquid nitrogen tank with a transfer hose.
- Pre-filled, RNase-free 2-ml bead tubes (e.g., MP Biomedicals™ Lysing Matrix S, cat # MP116925050)
- RNA extraction kit, such as Bio-Rad Aurum™ Total RNA Fatty and Fibrous Tissue Kit (cat # 7326830) or Qiagen RNeasy^®^ Lipid Tissue Kit (cat # 74804) and RNase-free DNase Set (cat # 79254)
- Chloroform, molecular biology grade (e.g., Thermo Scientific cat # J67241-AP)
- Ethanol, molecular biology grade (e.g., Thermo Scientific cat # T038181000)
- DEPC-treated water (e.g., Invitrogen™ cat # AM9920)
- RNase decontamination reagent (e.g., RNaseZap™, cat # AM9780)
- 5-mm^2^ cube silicone molds for embedding tissue (can be purchased from craft stores or Etsy shops specializing in supplies for making resin jewelry)
